# Community Health Educator’s Role on the 3D Team

**DOI:** 10.1093/geroni/igaa057.2681

**Published:** 2020-12-16

**Authors:** Alba Santiago

**Affiliations:** UConn Health, Farmington, Connecticut, United States

## Abstract

As a member of the 3D Team, the bilingual, bicultural Community Health Educator (CHE) addresses needs expressed by study participants related to social determinants of health. Clinical triggers generated by nurse practitioners (NPs) that lead to CHE referral include: social isolation and loneliness; lack of transportation access; lack of resources to sustain nutritional adequacy, purchase medications, and purchase assistive devices; and cultural and linguistic barriers that lead to lack of knowledge about community resources. To date, 50% of study participants randomized to receive 3D Team care have triggered referral to the CHE. In this presentation, the team CHE will provide details on the frequency of different needs expressed by study participants, how she utilizes an ever-growing community resource directory, and specific types of information and guidance she provides to address their expressed needs. Case studies will help illustrate ways in which CHE services have successfully provided assistance to study participants.

